# Head impacts sustained by male collegiate water polo athletes

**DOI:** 10.1371/journal.pone.0216369

**Published:** 2019-05-02

**Authors:** Nicholas J. Cecchi, Derek C. Monroe, Gianna M. Fote, Steven L. Small, James W. Hicks

**Affiliations:** 1 Department of Ecology and Evolutionary Biology, University of California Irvine, Irvine, California, United States of America; 2 Department of Neurology, University of California Irvine, Irvine, California, United States of America; 3 Department of Biological Chemistry, University of California Irvine, Irvine, California, United States of America; Cleveland Clinic, UNITED STATES

## Abstract

Water polo is a contact sport that is gaining popularity in the United States and carries a risk of repeated head impacts and concussion. The frequency and magnitude of sport-related head impacts have not been described for water polo. We aimed to compare patterns of empirically measured head impact exposure of male collegiate water polo players to patterns previously reported by a survey of current and former water polo athletes. Participants wore water polo caps instrumented with head impact sensors during three seasons of collegiate water polo. Peak linear acceleration (PLA) and peak rotational acceleration (PRA) were recorded for head impacts. Athlete positions were recorded by research staff at the occurrence of each head impact. Head impacts were sustained by athletes in offensive positions more frequently than in defensive and transition positions (246, 59.9% vs. 93, 22.6% vs. 72, 17.5%). 37% of all head impacts during gameplay were sustained by athletes playing the offensive center position. Impact magnitude (means ± SD: PLA = 36.1±12.3g, PRA = 5.0±2.9 krads/sec^2^) did not differ between position or game scenario. Among goalies, impact frequency and magnitude were similar between games (means ± SD: 0.54±.51 hits/game, PLA = 36.9±14.2g, PRA = 4.3±4.2 krads/sec^2^) and practices (means ± SD: 0.96±1.11 hits/practice, PLA = 43.7±14.5g, PRA = 3.9±2.5 krads/sec^2^). We report that collegiate water polo athletes are at risk for sport-related head impacts and impact frequency is dependent on game scenario and player position. In contrast, magnitude does not differ between scenarios or across positions.

## Introduction

Head impacts and concussions are common among athletes participating in a wide variety of sports [1,2]. Between 1.6 and 3.8 million sport-related concussions are estimated to occur annually in the United States, many of which go unreported [3]. In some cases, concussion leads to devastating neurological consequences [4], but head impact exposure, even in the absence of diagnosed concussion, may also have acute and chronic physiological and psychological effects [5–8]. The frequency and magnitude of head impacts in sports such as American football [9,10], lacrosse [11], soccer [12,13], and ice hockey [14] have received increasing attention due to the physical and aggressive nature of competition in these sports. Water polo is a rigorous, full-contact, and rapidly growing sport in the United States [15] that is associated with a risk of head and face injury [16–18] in which head impacts have not been studied *in vivo*. In a first-of-its-kind survey, water polo athletes reported sustaining an average 2.27 “serious blows to the head” imposed by both the ball and opposing athletes per game over the span of their career [16]. Game mechanics and commonly used strategies in water polo present unique risks for sport-related head impacts, but objective and prospective data validating these survey findings and clarifying sport-specific risk factors are needed.

Growing evidence suggests that impact magnitude, and not frequency alone, is important when considering the neurological effects of repeated sport-related head impacts [5,6,8]. Thus, we sought to use head-worn inertial sensors to monitor head impacts sustained by male collegiate water polo players. We expected to observe patterns previously reported in survey data: i) Goalies would incur more head impacts during practices than in games, and ii) athletes occupying the center positions on offense and defense would sustain more head impacts than those occupying other field positions [16].

## Methods

### Participants

Three cohorts of Division 1 NCAA Men’s Water Polo players wore impact sensors during official American Water Polo sanctioned games and practices throughout the 2015, 2016, and 2017 seasons. No athletes were diagnosed with a concussion during the monitoring period. All data collection activities were approved by the Institutional Review Board of the University of California, Irvine.

### Measurement methods

**Fig 1:**
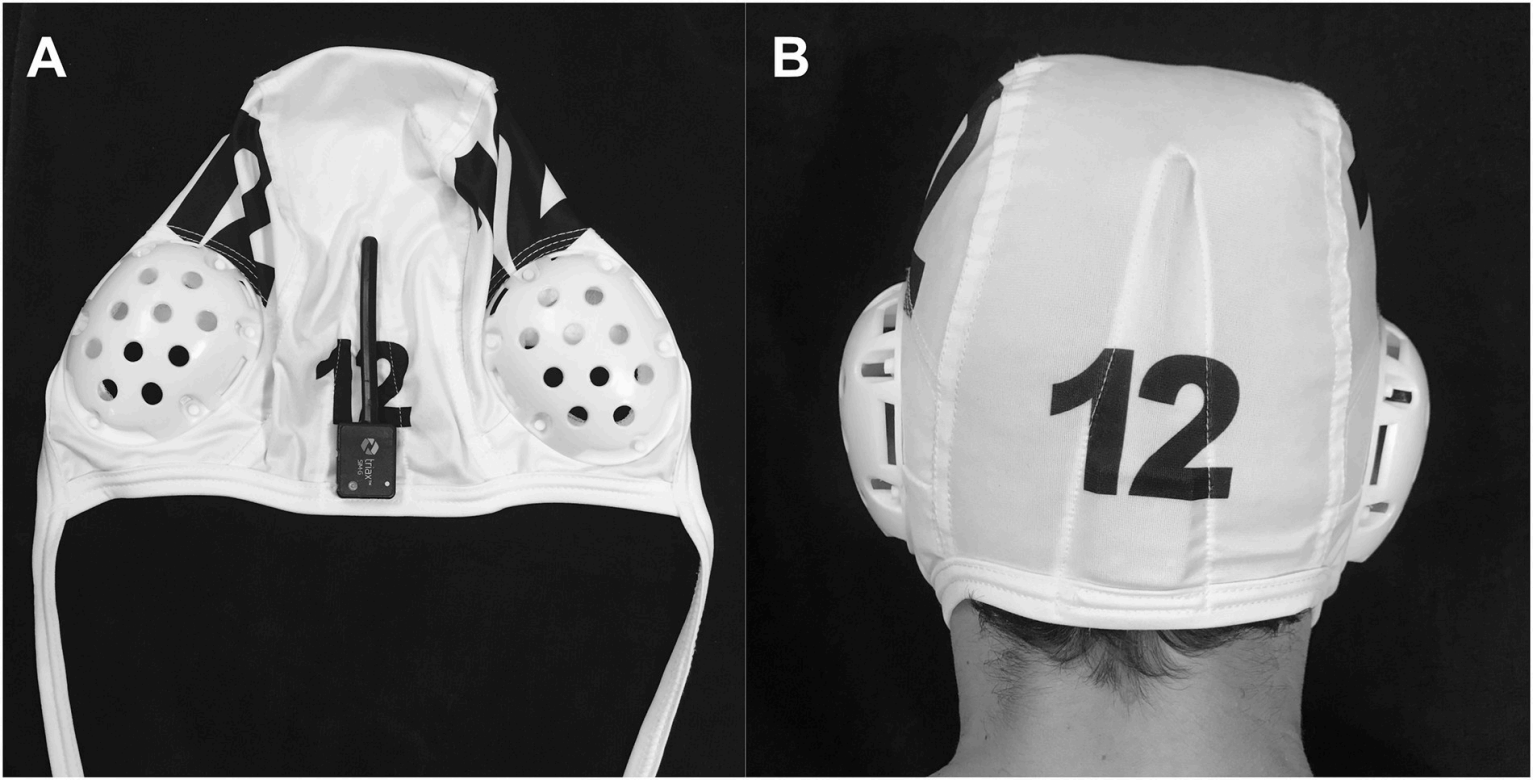
A) SIM-G placement in a modified water polo cap and B) Athlete wearing a modified water polo cap with SIM-G inserted.

Participants were fitted with SIM-G head impact sensors that relayed data in real-time to a sideline device (Triax Technologies; Norwalk, CT). Each SIM-G sensor has a 3-axis gyroscope, high-g 3-axis accelerometer, low-g 3-axis accelerometer, rechargeable lithium ion battery and 900 MHz radio, all contained within a waterproof plastic housing. Laboratory evaluations of the SIM-G suggest that the SIM-G accurately and consistently records peak linear and rotational accelerations when coupled tightly to the occipital protuberance [19,20]. SIM-G sensors were inserted into modified water polo caps, each of which was modified to include a Velcro pocket designed specifically to tightly house a SIM-G sensor that would rest on the occipital protuberance, with its antenna pointing upwards (Fig 1). Each participant was linked to a specific SIM-G sensor and wore the same sensor at each event for the duration of their participation. Both field players and goalies were monitored in games. Goalies were also monitored during practices. Impact data were collected across 23 games and 23 practices spanning the three American Water Polo seasons.

The SIM-G sensors recorded the peak linear acceleration (PLA) and peak rotational acceleration (PRA) associated with each head impact. The SIM-G sensors have a standard recording threshold of 16g, and thus only impacts registering a peak linear acceleration of 16g or greater were recorded. The sensors feature a complex algorithm that aims to distinguish hit-related head movement from commonly occurring non-impact transients (e.g., voluntary head movement, adjusting of headgear, sensor movements about the head) based on hit waveform topography.

Research staff monitored both the sideline device and athletes to validate the occurrence of head impacts (“hits”) as reported by the sensors. Notes were taken immediately following each impact reported by the sensors to identify false positives and record player positions for confirmed head impacts. Due to limitations in head impact sensor technology, instances in which an athlete’s cap was visibly uncoupled from the head or the athlete’s head could not be seen (e.g., when underwater) were marked as false positives. Games were recorded on video during the 2017 season to assist in identifying player positions and eliminating false positives.

### Data Processing

To reduce the number of false positives recorded by the SIM-G sensors, only data points that 1) passed through sensors’ algorithm as ‘real hits’ and 2) were visually verified by research staff on-site were included in statistical analyses. In 2017, false positives identified using video recordings of games were also removed from analysis.

**Fig 2:**
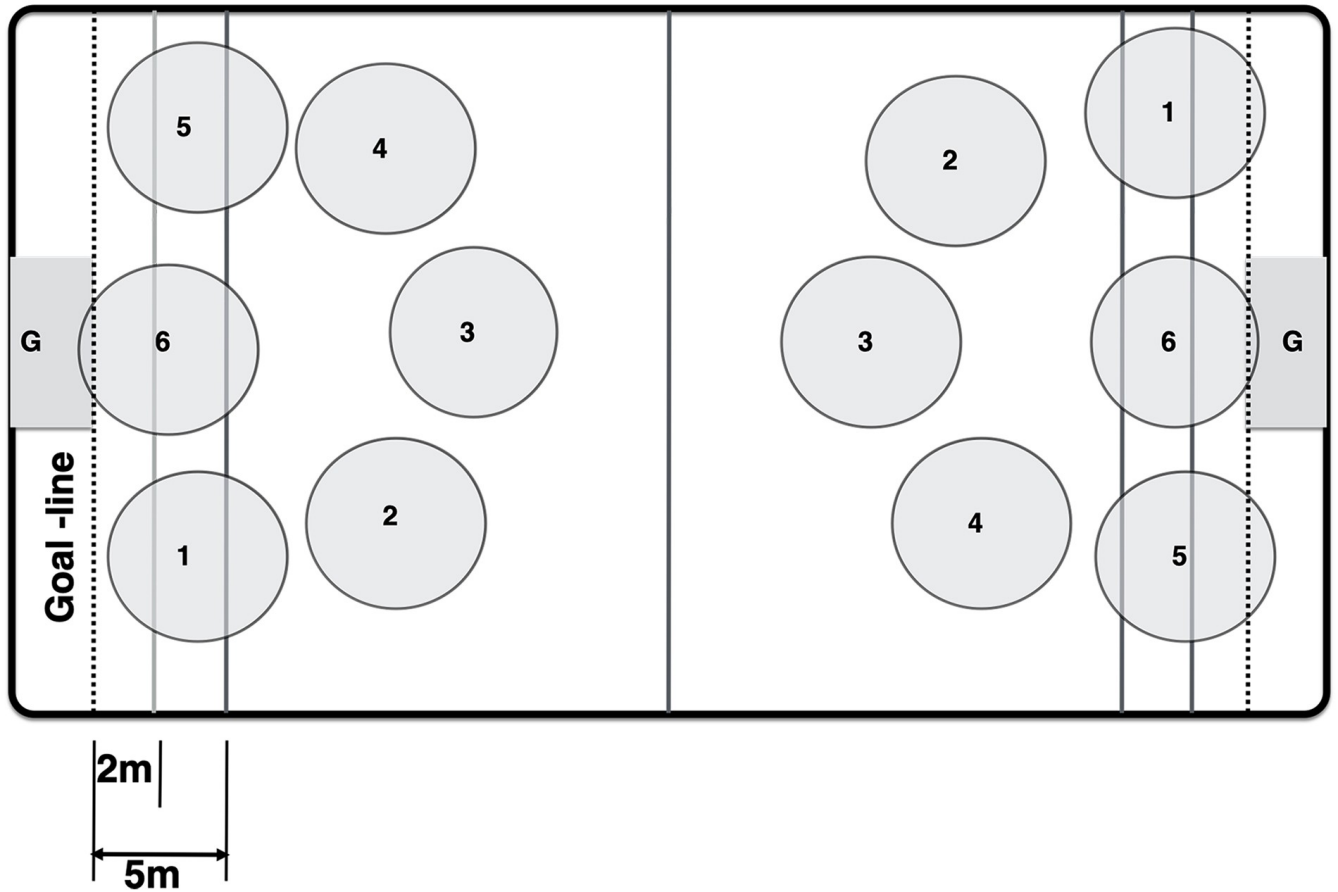
Locations of numbered player positions (1–6) and goalie (G) as observed in water polo games.

Player positions were initially divided into 16 categories based on game scenarios (offense, defense, or transition) and traditionally defined zones that form a rectangle around the goal (1–6) (Fig 2). Players occupying the 6 zone, which is centrally located and directly in front of the goal, are referred to as “centers”; the remaining zones, 1–5, which form a perimeter around the center, are referred to as “perimeter” positions. For purposes of this study, “transition” is defined as the period in which a player is not in a set offensive or defensive position, but rather, is moving from one side of the pool to the other, from offense to defense (OD) or defense to offense (DO). The three hits that occurred during the introduction of the ball at the beginning of a quarter (sprint) were also defined as occurring during ‘transition’. Data were aggregated using Microsoft Excel and figures and tables were created using Prism 7 (Graphpad Software Inc.; La Jolla, CA).

### Statistical analysis

Statistical analyses were performed using SPSS 24 (IBM; Armonk, NY). The null hypothesis that impact frequencies were equally distributed between player positions (n = 16) was tested using a Chi-square ‘goodness-of-fit’ test. A significant difference was decomposed by chi-square tests of the null-hypothesis that hits were equally distributed across player-by-position groupings and game scenarios based on patterns of risk revealed by Blumenfeld et al. [16] and common, sport-specific strategies. Bonferroni corrections for multiple comparisons were applied (p-value*6); corrected p-values are reported.

Separate Kruskal-Wallis analyses were used to test for differences in impact magnitudes (PLA, PRA) between player positions (n = 16). Separate Mann-Whitney U tests were used to test for differences in impact magnitudes (PLA, PRA) sustained by goalies between practices and games.

## Results

**Fig 3:**
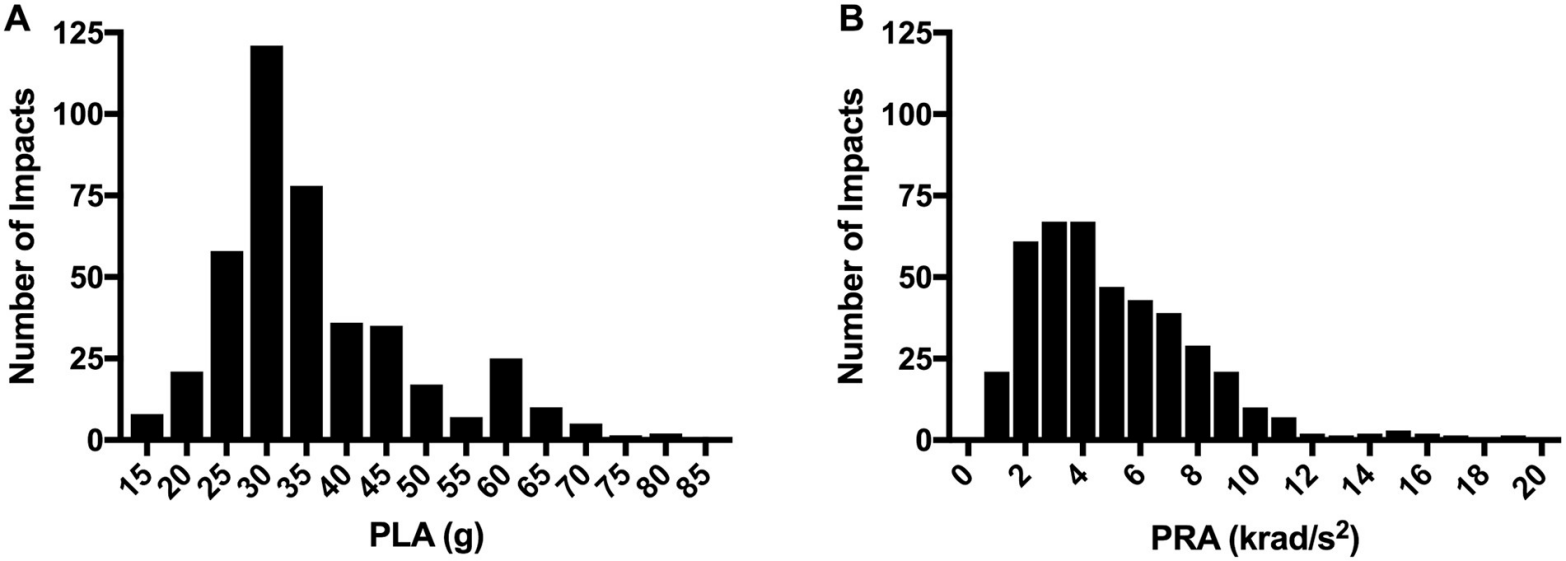
Frequency distribution of head impacts by magnitude of A, peak linear acceleration (PLA), and B, peak rotational acceleration (PRA).

Overall, 46.39% of all recorded impacts turned out to be false positives and are not included in these analyses. A total of 424 verified impacts recorded during games were included in the analysis. 85 verified impacts were recorded in 2015 (12 field players, 2 goalies; 7 games), 84 verified impacts in 2016 (14 field players, 1 goalie; 4 games), and 255 verified impacts in 2017 (22 field players, 3 goalies; 12 games). Hits during game play averaged 36.1±12.3g PLA and 5.0±2.9 krads/sec^2^ PRA (Fig 3).

There was a difference in impact frequencies sustained during gameplay across all player positions [X^2^(15) = 772.158, p <.001]. Field players sustained more hits on offense than on defense or during transition [X^2^(1)>69.053, p <.001], but impact frequency was no different between defensive and transition positions [X^2^(1) =.2673, p =.612]. Players in the offensive [X^2^(1) = 390.44, p <.0001] and defensive [X^2^(1) = 74.4, p <.0001] “center” positions sustained more impacts than athletes in field positions in the same game scenario (i.e., offense and defense, respectively). However, offensive perimeter players also sustained more impacts than defensive perimeter players [X^2^(1) = 13.696, p <.001]. Left offensive positions (zones 1 and 2) experienced more impacts than right offensive positions (zones 4 and 5) [X^2^(1) = 8.803, p =.038]. Impact PRA [X^2^(15) = 6.353, p =.973] and PLA [X^2^(15) = 24.282, p =.060] did not differ across player positions. Neither impact magnitudes [Mann-Whitney U <184.00, p >.169] nor frequencies [X^2^(1) = 2.314, p =.123] differed between games and practices among goalies. Data are summarized in Table 1.

**Table 1:** Summary of recorded head impacts sustained by player positions.

Player Position	No. of Recorded Head Impacts	Mean (SD) No. of Recorded Head Impacts Per Game	Mean (SD) PLA/Impact	Mean (SD) PRA/Impact
**Offensive Zone**				
1	23	1.0 (1.0)	33.1 (13.0)	5.3 (3.0)
2	25	1.1 (1.1)	36.3 (13.2)	5.0 (2.7)
3	18	0.8 (1.2)	42.7 (14.6)	4.8 (2.5)
4	11	0.5 (0.7)	34.1 (8.3)	4.7 (3.1)
5	12	0.5 (0.7)	34.3 (9.6)	4.4 (2.2)
6	157	6.8 (3.9)	36.1 (11.9)	4.9 (2.8)
*Total*	*246*	*10*.*7 (5*.*0)*	*36*.*2 (12*.*2)*	*4*.*9 (2*.*8)*
**Defensive Zone**				
1	9	0.4 (0.7)	34.0 (15.1)	4.6 (2.0)
2	10	0.4 (0.8)	38.0 (15.0)	5.2 (2.4)
3	12	0.5 (0.7)	36.7 (14.5)	5.9 (4.1)
4	10	0.4 (0.7)	29.5 (8.2)	5.4 (2.6)
5	5	0.2 (0.6)	31.4 (3.9)	4.7 (3.0)
6	47	2.0 (1.7)	38.2 (15.0)	5.4 (3.4)
*Total*	*93*	*4*.*0 (2*.*6)*	*36*.*3 (14*.*0)*	*5*.*3 (3*.*2)*
**Transition**				
DO	37	1.6 (1.8)	34.9 (8.5)	4.8 (2.4)
OD	32	1.4 (1.1)	37.1 (11.4)	5.5 (3.6)
Sprint	3	0.1 (0.5)	27.4 (3.0)	4.3 (2.6)
*Total*	*72*	*3*.*1 (2*.*3)*	*35*.*6 (9*.*9)*	*5*.*1 (3*.*0)*
**Goalies**				
Game	13	0.5 (0.5)	37.0 (14.2)	4.3 (4.2)
Practice	22	1.0 (1.1)	43.7 (14.5)	3.9 (2.5)

_DO = Defense to Offense; OD = Offense to Defense_

## Discussion

This is the first quantitative report of head impacts sustained by water polo athletes during competition and practice. In support of survey data from Blumenfeld et al. [16], we report that collegiate water polo carries a risk of head impacts, apart from any specific diagnosis of “concussion”. The physiological importance of such impacts, in the absence of overt concussion, remains unclear. Although repeated head impacts sustained by some American football players appear to have serious neurological consequences [7], a large knowledge gap remains regarding the physiological and neurological effects of head impact exposure in other sports. Our findings suggest that highly skilled, competitive, collegiate water polo players comprise a cohort of contact athletes for whom repetitive head impacts, apart from overt concussion, might represent a risk factor for physiological dysfunction and neurological sequelae. In the current work, we analyzed impacts by position, rather than by player, since field positions are constant despite fluctuating team membership and individual playing time across three seasons. Whether head impact frequency and magnitude can be attributed to personal playing styles and/or individual physiological characteristics (e.g., body weight, neck strength) remains unknown [21].

In support of our hypotheses, and consistent with survey data [16], we found that offensive and defensive center positions are at the greatest risk for sustaining head impacts relative to other field player positions. This could be attributed to a common strategy for field players to get the ball to the center to optimize scoring opportunities–centers often accrue the most goals. Centers typically face away from the goal (unlike other field players) to receive the ball. Thus, offensive centers face away from their defenders, potentially increasing the risk of striking each other’s heads while wrestling for position or engaging in a scoring attempt. We also found that head impacts were more common on the left side, relative to the right side, of the pool. This pattern is consistent with another common offensive strategy to create scoring opportunities on the left side of the pool that utilize right-handed athletes, who are more common than left-handed athletes. Therefore, more shooting and movement tends to occur on the left side of the pool relative to the right side of the pool.

We monitored goalies (but not other players) during practices because, unlike field players, goalies appear to have more head impacts during practices than during games [16]. To the contrary, our findings suggest that similar patterns of risk exist at this position in games and in practice. However, it is likely that these data underestimate head impact exposure during a season’s worth of practices since our ratio of practices to games (1:1) is much less than a true practice to game ratio. Headgears validated to attenuate head impact kinematics are commonly used in other contact sports. Unlike other contact and collision sports, the headgears currently used universally across water polo are not required to meet protective standards. It is possible that player safety could be improved by a water polo cap capable of attenuating head impact kinematics.

Although the current data include only male collegiate water polo athletes, it is expected that a similar pattern of head impact risk based on game scenario and player position is also present in female collegiate water polo. It has been well documented that in organized contact sports in which men and women play by the same rules, women are more likely to have symptomatic head impacts [22,23]. Whether this is due to different distributions of impact frequency and magnitude, or to sex-specific physiological or neurological determinants of tolerance, remains unclear. Furthermore, studies in other sports have identified different patterns of head impacts and concussion across levels of competition and developmental stage [13,24]. Extending these epidemiological findings to other levels of water polo competition could help explain the nature of head-impact risk across the lifespan.

Head inertial sensor technology is still evolving, and these sensors have been demonstrated to be particularly sensitive to false positives. Not all studies have reported false positive rates, either because they did not rely on signal filtering or failed to utilize stringent observational verification techniques [10,13]. Other groups that did verify impacts suggest higher false positive rates than we do [25,26], but this is plausibly due to our use of the manufacturer’s algorithm that filters non-impact transients, thus reducing the total number of impacts that need to be confirmed, which we believe more accurately reflects the way these sensors would be used by coaches and athletic trainers for team management and monitoring. However, this algorithm likely leads to false negatives, i.e., a number of true head impacts are missed, implying that the impact frequencies we observed are underestimates of true exposure. Since not all seasons were video recorded, we were unable to quantify or report on these possible false negative data. Independent of whether an algorithm is used to ‘pre-screen’ the raw data, our study adds to a growing body of evidence suggesting that with current sensor technology, human observation performed by individuals knowledgeable in the sport is necessary to confirm impacts reported by the sensors—either in real-time or using video recordings [27].

One limitation of the current study is a lack of inter-rater agreement statistics, as neither real-time nor video observers kept independent records. In the future, an agreement metric (i.e., intraclass correlation coefficient) could be used to empirically validate the use of video recordings in classifying false positives or negatives [27]. Survey data also suggest that patterns of head impact location and mechanism are position specific [16], and subsequent studies of head impacts in water polo should seek to characterize these patterns. Furthermore, it is not possible to draw strong conclusions about injury risk from the absolute values of impact magnitudes, a limitation shared across a large body of research that utilizes inertial sensors to quantify sport-related head-impact exposure.

## Conclusion

The frequency of head impacts sustained by male collegiate water polo players is scenario and position dependent, but impact magnitude is independent of scenario and position. Intercollegiate water polo athletes may represent a valuable cohort for studying the acute and chronic effects of repeated head impacts in sport to extend our knowledge of athlete physiology and neurology and to inform evidence-based policies to promote the safety of athletes and the benefits of sport.
